# Formal Comment on “Platelet-leucocyte interactions drive MMP-mediated tissue damage in tuberculosis” (Kirwan et al., 2026)

**DOI:** 10.1371/journal.ppat.1014628

**Published:** 2026-09-24

**Authors:** Qingqing Shan

**Affiliations:** Department of Respiration, Chengdu Integrated TCM & Western Medicine Hospital, Chengdu, China; New Jersey Medical School, UNITED STATES OF AMERICA

**Dear Editor,**

We read with interest the article by Kirwan and colleagues, who demonstrate that platelet-leucocyte interactions via P-selectin/PSGL-1 drive matrix metalloproteinase (MMP)-mediated tissue damage in tuberculosis (TB) [1]. The authors show that platelets infiltrate *M.tb*-infected tissues, form aggregates with monocytes and neutrophils, and promote secretion of MMP-1 and MMP-10, implicating the P-selectin/PSGL-1 axis as a host-directed therapeutic target. Although this pathway has not been directly tested in vivo, zebrafish models indicate that platelet inhibition enhances anti-mycobacterial immunity [2], and clinical data link low-dose aspirin to reduced stroke risk in tubercular meningitis [3], collectively supporting the broader rationale for anti-platelet interventions in TB.

As clinicians managing patients with pulmonary TB, we recognize the translational potential of targeting the P-selectin/PSGL-1 axis. However, we wish to raise several methodological and clinical considerations that may merit further discussion.

First, the exclusive use of platelets and monocytes from healthy volunteers for in vitro co-culture experiments may not fully capture the pathophysiology in TB patients. The authors demonstrate that platelets enhance MMP-1 and MMP-10 secretion from *M.tb*-infected monocytes, with effects mediated in part through P-selectin/PSGL-1 interactions. However, these experiments used cells from healthy donors with no known TB exposure [1]. In TB patients, chronic inflammatory exposure may precondition both platelets and monocytes, potentially altering their activation status, receptor expression profiles, and functional responses. Notably, the authors themselves observe that platelet aggregation responses to ADP are significantly reduced in TB patients compared to healthy controls (Fig 4a) [1], yet the mechanistic experiments used platelets from healthy donors with presumably normal aggregation capacity. It remains possible, therefore, that the observed P-selectin/PSGL-1-dependent effects may differ quantitatively or qualitatively in cells from TB patients. Future studies replicating key experiments using cells isolated from TB patients would help validate the mechanistic pathway in the relevant pathological context.

Second, the optimal timing of antiplatelet intervention during TB treatment warrants further consideration. The authors report that platelet aggregation in TB patients is reduced at diagnosis and continues to decline during the first two weeks of anti-TB therapy before normalizing by eight weeks (Fig 5a-b) [1]. This temporal pattern raises questions about whether platelets become “exhausted” or consumed during active disease. If platelets are functionally attenuated at diagnosis, adjunctive antiplatelet agents might have limited additional benefit. However, the correlation data showing that P-selectin expression (associated with inflammation) correlates poorly with GPIIb/IIIa activation (associated with aggregation/thrombosis) (Fig 8) [1] suggests that pro-inflammatory platelet functions may be dissociated from aggregation capacity. This dissociation is consistent with P-selectin’s established role as a cell adhesion receptor that can be upregulated independently of thrombotic pathways [4]. Therefore, anti-inflammatory strategies targeting P-selectin might retain efficacy even when aggregation is reduced.

Notably, several recent clinical studies have explored adjunctive antiplatelet therapy in TB. A meta-analysis of randomized controlled trials in tubercular meningitis found that adjunctive low-dose aspirin significantly reduced stroke risk (RR: 0.56; 95% CI: 0.33-0.95) without increasing bleeding events. However, no mortality benefit was demonstrated [3]. Preclinical evidence from zebrafish models shows that thrombocyte inhibition using glycoprotein IIb/IIIa inhibitors reduces mycobacterial burden. This supports a detrimental role for infection-induced platelet activation [2]. These findings collectively suggest that antiplatelet agents may have a role as host-directed therapy in TB. However, the optimal timing and patient selection criteria remain to be defined.

Third, the effect of standard anti-TB drugs on platelet function and bone marrow reserve warrants consideration. Several anti-TB medications, particularly linezolid, are associated with myelosuppression including thrombocytopenia [5]. A recent multicenter retrospective cohort study in Uganda reported that among 412 rifampicin-resistant TB patients receiving linezolid-based regimens, 14.8% developed thrombocytopenia and 13.8% developed leukopenia [5]. Although standard first-line anti-TB drugs (rifampicin, isoniazid) have been associated with hematological effects in some cases, this is less well-defined than with second-line agents such as linezolid [6]. Nevertheless, for a subset of high-risk patients—such as those receiving linezolid-containing regimens for drug-resistant TB, individuals with pre-existing myelosuppression, or those with limited bone marrow reserve (e.g., post-hematopoietic stem cell transplant recipients or patients with advanced HIV)—the addition of anti-platelet agents may warrant a careful risk-benefit assessment [7]. This is particularly relevant given that several anti-TB drugs (including rifampicin, isoniazid, and linezolid) can independently affect platelet counts and function. Moreover, drug-drug interactions between antiplatelet agents and anti-TB medications add further complexity to treatment. Specifically, rifampicin, a potent inducer of cytochrome P450 enzymes, can alter the metabolism of antiplatelet drugs such as clopidogrel and warfarin. This pharmacokinetic interaction could potentially reduce antiplatelet efficacy or increase bleeding risk [8], a factor that should be considered if such adjunctive therapies are pursued clinically.

Fourth, while the authors demonstrate that platelet supernatant increases MMP secretion from *M.tb*-infected monocytes (Fig 1d) [1], the specific soluble factors responsible remain to be identified. The authors acknowledge that “contact-independent mechanisms must also be involved,” but do not investigate which platelet-derived soluble mediators drive this effect. Potential candidates include soluble P-selectin (released during platelet degranulation), TGF-β (of which platelets are a major circulating source), platelet factor 4 (PF4/CXCL4), or other chemokines stored in platelet alpha granules. Identifying these factors may reveal additional therapeutic targets. Some of these may be more amenable to pharmacological intervention than blocking cell-cell contact. Given that a significant proportion of circulating TGF-β originates from platelets and has been implicated in post-TB lung fibrosis [9], this represents a promising avenue for further investigation.

Despite these considerations, we commend Kirwan and colleagues for advancing our understanding of platelet biology in TB and identifying P-selectin/PSGL-1 as a potential therapeutic target. The dissociation between pro-inflammatory and pro-thrombotic platelet functions (Fig 8) [1] is particularly noteworthy. This suggests that targeting P-selectin might reduce tissue damage without substantially compromising hemostasis. Future studies examining the interplay between anti-TB drug toxicity—particularly in patients on linezolid-based regimens—and platelet-targeted host-directed therapies may help guide clinical trial design and patient selection for adjunctive anti-platelet strategies in TB.

We hope these observations contribute to the ongoing discussion regarding optimal strategies to translate these mechanistic insights into clinical practice.

Sincerely,

Qingqing Shan

Chengdu Integrated TCM & Western Medicine Hospital
